# Prognostic nomograms for patients with resectable hepatocelluar carcinoma incorporating systemic inflammation and tumor characteristics

**DOI:** 10.18632/oncotarget.13038

**Published:** 2016-11-03

**Authors:** Junyi Shen, Linye He, Chuan Li, Tianfu Wen, Weixia Chen, Changli Lu, Lvnan Yan, Bo Li, Jiayin Yang

**Affiliations:** ^1^ Department of Liver Surgery and Liver Transplantation Center, West China Hospital, Sichuan University, Chengdu, China; ^2^ Department of Radiology, West China Hospital, Sichuan University, Chengdu, China; ^3^ Department of Pathology, West China Hospital, Sichuan University, Chengdu, China

**Keywords:** nomogram, prognosis, HCC, hepatectomy

## Abstract

**Background:**

The model to predict the prognosis of resectable hepatocelluar carcinoma (HCC) has not been determined.

**Methods:**

Predictors were selected using Cox model. Nomograms were generated in the training set and validated in the validation set. The predictive ability of the nomogram was determined by concordance index and calibration curve.

**Results:**

Independent factors for overall survival including alpha-fetoprotein level (hazard ratio (HR):1.292), tumor size (HR:1.092), tumor number (HR:1.472), microvascular invasion (HR:1.660), neutrophil to lymphocyte count ratio (NLR) (HR:1.428), major vascular invasion (HR:2.485) and satellite lesions(HR:1.392) were selected into the nomogram for survival. The c-index in the training set and validation set were 0.767 and 0.719, respectively, which were statistically higher than those of the four conventional staging systems.(Barcelona Clinic Liver Cancer: 0.644 and 0.609; the seventh American Joint Committee on Cancer: 0.678 and 0.674; Cancer of the Liver Italian Program: 0.692 and 0.648; Hong Kong Liver Cancer: 0.689 and 0.639, *p* < 0.001 for all). A nomogram for predicting 3- and 5-year recurrence free survival was generated with the c-index of 0.746 for the training set and 0.718 for the validation set, respectively.

**Conclusions:**

We have generated nomograms predicting prognosis for HCC treated by hepatectomy with a higher predictive power.

## INTRODUCTION

Hepatocelluar carcinoma (HCC) in china alone accounts for appropriate 50% of the deaths from liver cancer worldwide due to high presence of Hepatitis B virus (HBV) infection. [1] Surgical resection is widely accepted for curative therapy. Small HCC with well preserved liver function is suitable for hepatectomy with 5-year survival rate of 70–81%. [2, 3] For patients with intermediate-advanced stage HCC, hepatectomy is not recommended based on current stage systems due to high recurrence rate, recent studies from western and eastern shown that surgical resection for some selected patients with intermediated-advanced stage HCCs could bring more survival benefits than transarterial chemoembolization (TACE). [4–6] With more patients suitable for hepatectomy, the model to predict all resectable HCCs but not only early stage HCC or specific stage HCCs was necessary. [7, 8] Liu et al. had proposed one model which had excellent discriminating power about prognosis of patients from early to advanced stages of HCC. [9] We believed that, for resectable, HCCs, the prediction power of nomograms with full consideration of pathological findings or/and some promising inflammation index might increase.

The conventional staging systems mainly focused on tumor status, such as tumor size, tumor number and vascular invasion. [10] They had some limitation in predicting the prognosis of HCC after surgery because the current staging systems put insufficient attention on pathological findings, such as microvascular invasion (MVI), satellite lesions, and acceptable risk factors like platelet-to-lymphocyte ratio (PLR) or/and neutrophil-to-lymphocyte ratio (NLR). For example, MVI representing tumor behavior had been demonstrated as an extremely important factor associated with the prognosis of HCCs. [11, 12] Only the seventh American Joint Committee on Cancer (AJCC) system had included this factor to distinguish the prognosis. Similarly, satellite lesion negatively impacting the prognosis was neglected in the conventional staging systems. [13, 14]. Besides, in most investigations, tumor size itself as continuous variable was included as a categorical variable and tumor number were simply classified as single and multiple HCCs. [15–18] Previous studies showed patients with tumor number more than 3 might present poor prognosis compared to patients with 1–3 tumors after surgery. [19] We hypothesized model incorporating tumor size as continuous variable might accurately predict the prognosis since tumor size was closely related with risk factors, such as MVI and satellite lesion. [20].

Recently, systematic inflammation index, such as PLR and NLR, has been focused by many investigators and demonstrated to be an independent risk factor associated with cancerous prognosis. [21–23] Inflammation closely correlated with cancer progress [24–26] In clinical, neutrophil counts, platelet counts and lymphocyte counts might reflect the host immune and inflammation status and thus indicate tumor progress through different combinations. [21] Unfortunately, they are few predictive models including these simple and effective factors.

Nomogram is a method that could assign relative risk scores to each predictor according to its contribution to the prognosis. Based on total points of individual, it could predict the probability of overall survival (OS) and recurrence free survival (RFS) after hepatectomy. Recently, many investigators successfully evaluate risk scores to more accurately predict the outcomes in various cancers including HCC. [27–29]. For resectable HCCs after hepatectomy, model incorporating all the available risk factors might increase power in predicting the prognosis of HCCs after hepatectomy.

The purpose of the current study is (1) to construct clinically useful nomograms with full consideration of tumor status and systematic inflammation to predict OS and RFS for resectable HCC after hepatectomy; (2) to validate the nomograms in the validation set; (3) to compare its predictive power with current popular staging systems.

## RESULTS

### Baseline characteristics in training set and validation set

A total of 777 subjects were included in the current study with 618 in the training set and 159 subjects in the validation set. The baseline characteristics of the two cohorts in all study subjects are shown in Table 1. In the training set, there were 150 patients (24.3%) with age > 60 year and 541 male patients.540 patients (87.4%) had HBV infection. The average tumor size was 6.7 cm ranging from1.2 cm to 25.0 cm. There were 478 patients with solitary HCC, 82 patients (13.3%) with two tumors and 58 patients (9.4%) with more tumors. Major vascular invasion was noted in 98 patients (15.9%) and MVI in 342 (37.5%). Satellite lesions were available for 96 patients (15.5%). In validation set, the distribution of these characteristics is almost similar to the training set (Table 1).

**Table 1:** Clinicopathological characteristics in the training set and validation set

		Training set	Validation set
		*n* = 618	*n* = 159
age > 60 y		150 (24.3)	40 (25.2)
Gender (male/female)		541/77	138/21
Positive HBsAg		540 (87.4)	140 (88.1)
Positive HBV load		292 (47.2)	80 (50.3)
AFP > 400 ng/ml		289 (46.8)	79 (49.7)
Differentiation	poor	276 (44.7)	65 (40.9)
	well-moderate	342 (55.3)	94 (59.1)
Satellite lesion		96 (15.5)	20 (12.6)
MVI		232 (37.5)	62 (39.0)
Tumor number	one	478 (77.3)	125 (78.6)
	two	82 (13.3)	17 (10.7)
	more	58 (9.4)	17 (10.7)
Tumor size		6.7 ± 3.7	7.3 ± 3.5
Ishak score	5–6	399 (64.6)	97 (61.0)
	0–4	219 (35.4)	62 (39.0)
Major vascular invasion		98 (15.9)	17 (10.7)
Positive surgical margin		13 (2.1)	3 (1.9)
Lg10ALT		1.6 ± 0.3	1.6 ± 0.2
Lg10AST		1.6 ± 0.2	1.6 ± 0.2
ALB		41.4 ± 4.4	40.8 ± 4.0
TBIL		15.2 ± 6.9	14.9 ± 6.7
PLR	≥ 111	224 (36.2)	67 (42.1)
	< 111	394 (63.8)	92 (57.9)
NLR	≥ 3	197 (31.9)	49 (30.8)
	< 3	421 (68.1)	110 (69.2)
Surgery type	Extend	151 (24.4)	46 (28.9)
	major	196 (31.7)	54 (34.0)
	minor	271 (43.9)	59 (37.1)
Blood transfusion		50 (8.1)	13 (8.2)
recurrence site	Intra-hepatic	360 (58.3)	93 (58.5)
	Extra-hepatic	78 (12.6)	18 (11.3)
recurrence treat	salvage LT	7 (1.1)	1 (0.6)
	re-resection	44 (7.1)	5 (3.1)
	radiofrequency ablation	34 (5.5)	8 (5.0)
	TACE	165 (26.7)	45 (28.3)
	pallitive therapy	189 (30.6)	53 (33.3)

HBsAg hepatitis B virus antigen; AFP alpha-fetoprotein, HBV-DNA hepatitis B virus DNA; MVI microvascular invasion, TBIL total bilirubin AST aspartate aminotransferase, ALT alanine aminotransferase; ALB albumin; TBIL total bilirubin; PLR platelet to lymphocyte ratio; NLR neutrophil to lymphocyte ratio; LT liver transplantation; TACE transcatheter arterial chemoembolization.

### Follow-up treatments and predictors of OS

During follow-up, 548 patients (70.9%) suffered from postoperative recurrence with 453 patients with intrahepatic recurrence and 96 patients with extrahepatic recurrence. Of the 548 patients with recurrence, eight patients received liver transplantation, 49 patients received resection, 42 patients received radiofrequency ablation, 210 patients received TACE and 242 received palliative therapy. In univariate analysis of the training set, age, gender, AFP, satellite lesion, MVI, tumor number, tumor size, major vascular invasion, positive surgical margin, alanine transaminase (ALT), aspartate transaminase (AST), albumin (ALB), PLR, NLR, surgery type were potential risk factors for postoperative On multivariable analysis, alpha-fetoprotein (AFP) (*P* = 0.031 hazard ration (HR) 1.292 95% confidence interval (CI) 1.024–1.629), NLR (*p* = 0.004 HR 1.428 95% CI 1.123–1.817), tumor size (*p* < 0.001 HR 1.092 95% CI 1.058–1.127), tumor number (*p* < 0.001 HR 1.350 95% CI 1.146–1.589), satellite lesions (*p* = 0.028 HR 1.392 95% CI 1.036–1.869), MVI (*p* < 0.001 HR 1.660 95% CI 1.304–2.113) and surgery type (*p* = 0.005 HR 1.250 95% CI 1.071–1.459) showed prognostic power (Table 2).

**Table 2:** Variables associated with OS according to the Cox proportional hazard model

Variables	Univariate analysis			Multivariate analysis		
	*p*	HR	95% CI	*p*	HR	95% CI
age > 60 y	0.032	0.747	0.572–0.974			
Gender (male/female)	0.039	1.423	1.030–1.964			
Positive HBsAg	0.178					
Positive HBV load	0.221					
AFP > 400 ng/ml	< 0.001	1.879	1.508–2.342	0.031	1.292	1.024–1.629
Differentiation (poor/moderate–well)	0.001	1.443	1.160–1.796			
Satellite lesion	< 0.001	2.580	1.978–3.367	0.028	1.392	1.036–1.869
MVI	< 0.001	2.593	2.077–3.237	< 0.001	1.660	1.304–2.113
Tumor number (one/two/more)	< 0.001	1.472	1.260–1.721	< 0.001	1.350	1.146–1.589
Tumor size	< 0.001	1.148	1.120–1.176	< 0.001	1.092	1.058–1.127
Ishak score (5–6/0–4)	0.829					
Major vascular invasion	< 0.001	4.728	3.550–6.298	< 0.001	2.485	1.813–3.407
Positive surgical margin	0.001	2.990	1.532–5.835			
Lg10ALT	0.043	1.420	1.011–1.994			
Lg10AST	< 0.001	2.447	1.596–3.752			
ALB	0.050	0.974	0.949–1.000			
TBIL	0.924					
PLR (> 111/ ≤ 111)	< 0.001	1.734	1.388–2.167			
NLR (> 3/ ≤ 3)	< 0.001	1.939	1.549–2.427	0.004	1.428	1.123–1.817
PT	0.139					
Surgery type (extend/major/minor)	< 0.001	1.689	1.474–1.936	0.005	1.250	1.071–1.459
Blood transfusion	0.110					

HR hazard ration; CI confidence interval; HBsAg hepatitis B virus antigen; AFP alpha-fetoprotein, HBV-DNA hepatitis B virus DNA; MVI microvascular invasion, TBIL total bilirubin AST aspartate aminotransferase, ALT alanine aminotransferase ; ALB albumin; TBIL total bilirubin; PLR platelet to lymphocyte ratio; NLR neutrophil to lymphocyte ratio; PT prothrombin time.

### Construction and validation of the nomogram

**Figure 1:**
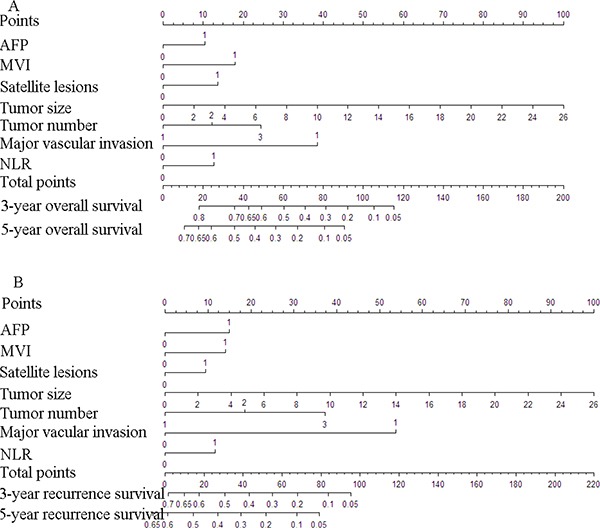
Nomograms to predict probability of 3- and 5-year over survival (**A**) and recurrence free survival (**B**) for HCC treated by hepatectomy To calculate the probability of postoperative survival, we first draw a vertical line to the points scale to get the value for each factor and then sum up all the individual values. We finally draw a vertical line from the total points scale to the probability at the probability at the year 3 line to obtain 3-year survival rate and at the year 5 line to obtain 5-year survival rate. AFP: 0 = < 400 ng/ml, 1 = > 400 ng/ml; MVI: 0 = negative,1 = positive; Satellite lesions: 0 = negative,1 = positive; Tumor number: 1=one,2=two,3=more; Major vascular invasion: 0 = negative,1 = positive; NLR: 0 = < 3,1 = ≥ 3.

**Figure 2:**
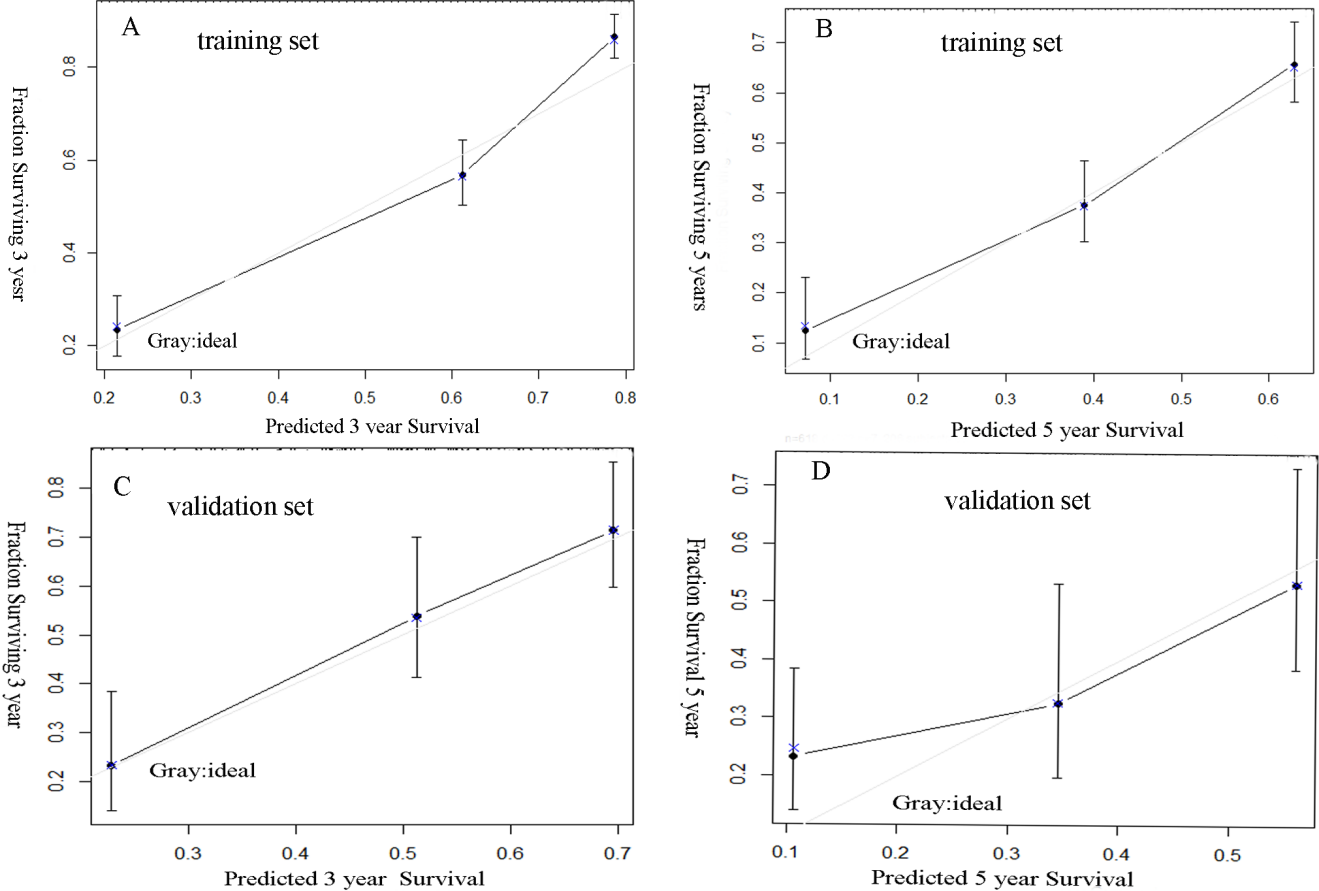
Good calibration of the nomogram in the training and validation set The nomogram predicted the probabilities of postoperative 3 years survival (**A**), 5 years survival (**B**) in the training set and 3 years survival (**C**), 5 years survival (**D**) in the validation set.

A nomogram to predict OS of patients with HCC following surgical resection is shown in Figure 1A. Spearman correlation indicated that surgery type versus tumor size correlated significantly (*r* = 0.402, *P* < 0.001). Except for surgery type, the nomogram included other seven variables associated with OS in multivariable analysis. Each factor in the nomogram was assigned a weighted number of points, and the sum of points for each patient was in accordance with a specific predicted 3- and 5-year OS. A higher score predicted worse prognosis (Figure 1).The model demonstrated good accuracy for predicting overall survival rate of HCC after hepatectomy, with a c-index of 0.767 (95% CI 0.742–0.792). The bootstrapped calibration plot for the prediction of 3-year and 5-year OS is shown in Figure 2A and 2B. The calibration plots revealed good prediction of 3- and 5-year OS.

In the validation set, the C-index of the nomogram for predicting OS was 0.719 (95% CI, 0.671 to 0.767), and a calibration curve showed good agreement between prediction and observation in the probability of 3-year and 5-year survival (Figure 2C and 2D).

Similarly, based on the multivariate analysis, the seven risk factors included in the OS nomogram were indentified predictors of recurrence free survival (Table 3). A nomogram to predict 3- and 5-year RFS of patients with HCC following surgical resection is shown in Figure 1B. The model demonstrated good accuracy for predicting RFS rate of HCC after hepatectomy, with a c-index of 0.746 (95% CI, 0.723 to 0.768). In the validation set, the C-index of the nomogram for predicting RFS was 0.718 (95% CI, 0.670 to 0.766)

**Table 3:** Variables associated with RFS according to the Cox proportional hazards model

Variables	Univariate analysis			Multivariate analysis		
	*p*	HR	95% CI	*p*	HR	95% CI
age > 60 y	0.041	0.794	0.636–0,991			
Gender (male/female)	0.003	1.615	1.715–2.221			
Positive HBsAg	0.169					
Positive HBV load	0.179					
AFP > 400 ng/ml	< 0.001	1.843	1.526–2.227	0.001	1.476	1.162–1.874
Differentiation (poor/moderate-well)	< 0.001	1.406	1.164–1.697			
Satellite lesion	< 0.001	2.271	1.782–2.895	0.001	1.867	1.135–2.576
MVI	< 0.001	2.319	1.913–2.811	0.005	1.433	1.116–1.839
Tumor number(one/two/more)	< 0.001	1.553	1.355–1.779	0.001	1.287	1.090–1.519
Tumor size	< 0.001	1.119	1.095–1.144	0.032	1.041	1.004–1.079
Ishak score (5–6 / 0–4)	0.639					
Major vascular invasion	< 0.001	4.663	3.618–6.011	< 0.001	3.426	2.126–5.521
Positive surgical margin	< 0.001	3.012	1.649–5.500			
Lg10ALT	0.21					
Lg10AST	0.021	1.561	1.070–2.278			
ALB	0.032	0.976	0.954–0.998			
TBIL	0.994					
PLR (> 111 / ≤ 111)	< 0.001	1.55	1.278–1.881			
NLR ( >3 / ≤ 3)	< 0.001	1.731	1.422–2.107	0.003	1.469	1.135–1.902
PT	0.023	1.149	1.020–1.295			
Surgery type (extend/major/minor)	< 0.001	1.584	1.411–1.779			
Blood transfusion	0.291					

Abbreviations as Table 2.

### Survival analysis based on current staging system

**Figure 3:**
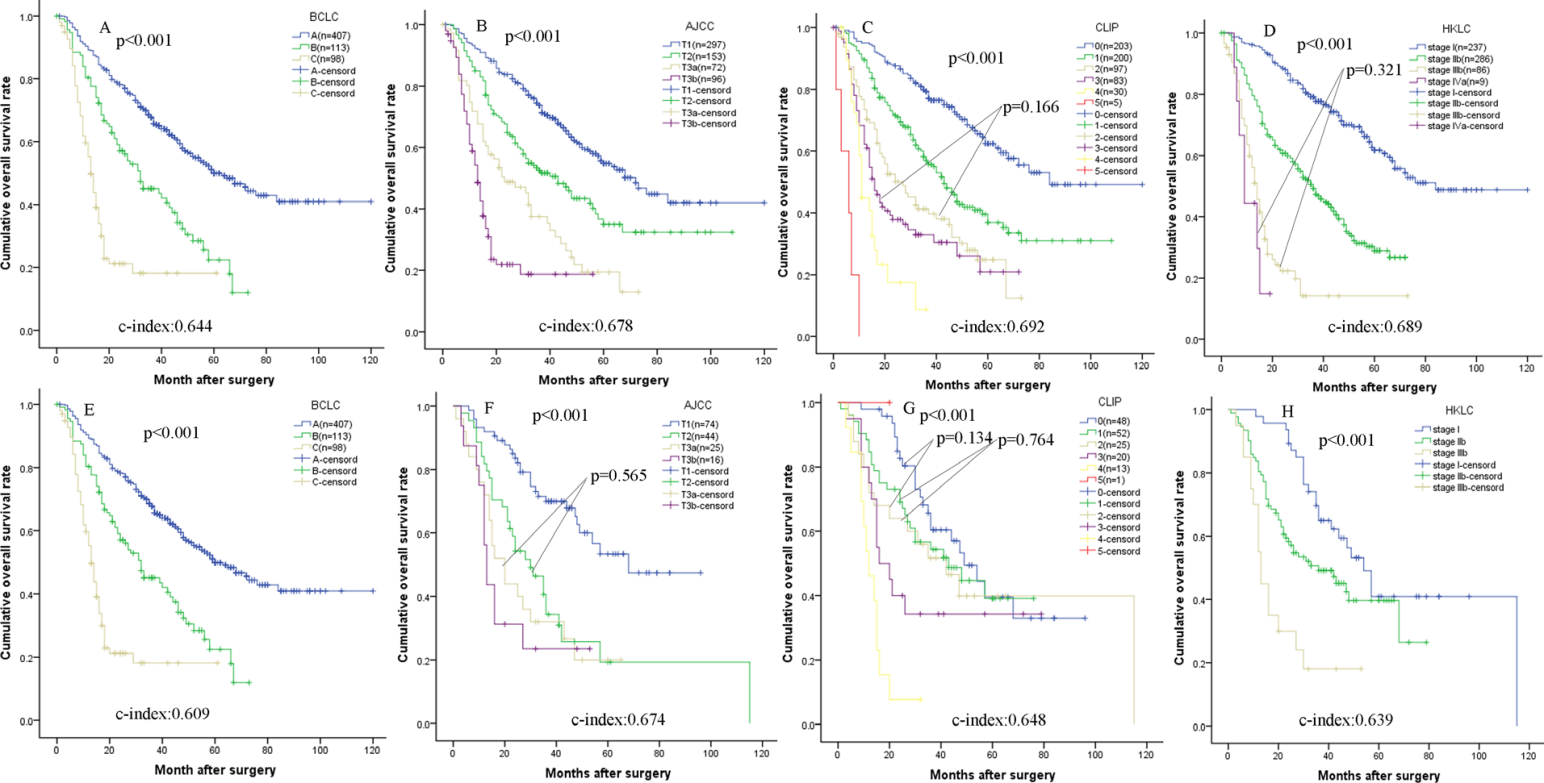
Kaplan-Meier survival curves of the training set (**A**) Barcelona Clinic Liver Cancer (BCLC) staging system; (**B**) American Joint Committee on Cancer (AJCC) seventh edition; (**C**) Cancer of the Liver Italian Program (CLIP) staging system; (**D**) Hong Kong Liver Cancer (HKLC) staging system) and the validation set ((**E**) Barcelona Clinic Liver Cancer (BCLC) staging system; (**F**) American Joint Committee on Cancer (AJCC) seventh edition; (**G**) Cancer of the Liver Italian Program (CLIP) staging system; (**H**) Hong Kong Liver Cancer (HKLC) staging system) classified by different staging systems.

In the current study, Barcelona Clinic Liver Cancer (BCLC) system, the seventh AJCC system, Cancer of the Liver Italian Program (CLIP) system and Hong Kong Liver Cancer (HKLC) system were used to classify all the patients. The results were shown in Table 3. The survival analysis stratified by BCLC system, the seventh AJCC system, CLIP system and HKLC system were present in Figure 3 and Table 4.

**Table 4:** Overall survival analysis stratified by staging systems in the training set and validation set

Staging system		Training set				Validation set			
		*n* = 618	1-year	3-year	5-year	*n* = 159	1-year	3-year	5-year
BCLC system	A	407 (65.9)	89.2	67.7	49.9	110 (69.2)	88.2	56.7	41.4
	B	113 (18.3)	80.4	45.1	22.4	32 (20.1)	78.1	39.8	23.9
	C	98 (15.9)	55.3	18.2	-	17 (10.7)	52.9	22.1	-
AJCC system	T1	297 (48.1)	92.2	73.7	54.8	74 (46.5)	91.9	70.0	53.3
	T2	153 (24.8)	85.0	51.6	35.0	44 (27.7)	81.8	34.3	19.3
	T3a	72 (11.7)	71.8	37.5	19.4	25 (15.7)	72.0	32.0	20.0
	T3b	96 (15.5)	54.3	18.8	-	16 (10.1)	56.3	23.4	−95.0
CLIP system	0	203 (32.8)	95.0	79.3	62.4	48 (30.2)	97.9	60.4	44.5
	1	200 (32.4)	89.5	56.3	36.8	52 (32.7)	84.6	54.4	39.1
	2	97 (15.7)	73.5	41.3	24.9	25 (15.7)	56.0	51.7	39.9
	3	83 (13.4)	62.2	33.0	20.9	20 (12.6)	75.0	34.3	-
	4	30 (4.9)	44.8	8.7	-	13 (8.2)	46.2	7.7	-
	5	5 (0.8)	-	-	-	1 (0.6)	-	-	-
HKLC system	1	237 (38.3)	96.2	79.2	61.7	47 (29.6)	97.9	65.0	40.9
	2b	286 (46.2)	79.6	49.2	29.0	92 (57.9)	80.4	49.2	39.7
	3b	86 (13.9)	56.1	14.2	-	20 (12.6)	55.0	18.0	-
	4a	9 (1.5)	44.4	-	-	0 (0.0)	-	-	-

BCLC Barcelona Clinic Liver Cancer staging system; AJCC American Joint Committee on Cancer seventh edition; CLIP Cancer of the Liver Italian Program staging system; HKC Hong Kong Liver Cancer staging system.

### Comparsion of the nomogram and current staging system

The c-index for OS in the training set and the validation set were 0.767 (95% CI 0.742–0.792) and 0.719 (95% CI 0.671–0.767), respectively. In the training set, There were significant differences in the C-indices between the nomogram and other staging systems (0.767 vs. BCLC: 0.644; the seventh AJCC system: 0.678; CLIP: 0.692 and HKLC system; 0.688; *P* < 0.001 for all, respectively), and all C-indices were significantly lower than that of the nomogram. In the validation set, There were also significant differences in the C-indices between the nomogram and other staging systems (0.719 vs. BCLC: 0.609; the seventh AJCC system: 0.674; CLIP: 0.648 and HKLC system; 0.639; *P* < 0.001 for all, respectively), and all c-indices were significantly lower than that of the nomogram.

## DISCUSSION

Currently, surgical resection remains the curative treatment modality for hepatocelluar carcinoma without metastasis. [1, 5] Hepatectomy was considered when all tumors on preoperative imaging studies could technically be resected and preserved liver function is well. Recently, many hepatobiliary institutions, including western and eastern liver cancer centers, advocate hepatectomy to treat HCC outside the Milan criteria. [5, 6] Personal prognostic prediction is essential to guild post-operative additional treatment and postoperative counseling. The prognosis of HCC patients varies greatly according the tumor status, liver function and performance status. In clinical, for those with resectable HCCs, they almost had Child-Pugh status A even they had intermediate-advanced stage HCCs. Consistently, in this study, Child-Pugh status or liver cirrhosis did not impact the prognosis after hepatectomy. [5, 6, 30] Surgery type was considered as predictor of prognosis in the Cox model. However, according to the pearson correlation analysis, surgery type and tumor size correlated closely. In clinical practice, patients with large tumor size might face that more liver tissue were be removed in order to eliminate tumor. We therefore select tumor size for analysis, namely tumor size and then construct this nomogram by combination of tumor characteristics and systematic inflammation.

Tumor characteristics in our nomogram included AFP level, tumor size, tumor number, MVI, major vascular invasion and satellite lesions. In consistent with previous study, all the variables were validated as independent risk factors in our large sample size. [5, 31] The cutoff value of AFP was 400 ng/ml as previous study showed. [32] AFP > 400 ng/ml was assigned score of 10.4 points for OS prediction and 15 points for RFS prediction Tumor size was ranging from 1.2cm to 26.0cm and tumor size with 26.0 cm was assigned score of 100 points for OS prediction and RFS prediction. Tumor number was classified as one, two and more with scores of 0, 12.2 and 24.4 points, respectively. The MVI, major vascular invasion and satellite lesions obtained score of 18.0 points, 38.5 points and 13.6 points, respectively for OS prediction and 14.1 points, 53.8 points and 9.4 points, respectively for RFS prediction. In contrast to many previous studies, we included tumor size as continuous variable because various cutoff of tumor size are validated as risk factor for HCC. [31, 33] The higher the points were, the larger the tumor size was. Our previous study suggested tumor number > 3 decreased the prognosis after hepatectomy. [19] In our nomogram, tumor number > = 3 had the max score of about 25.0 points for OS prediction and 40 points for RFS prediction. Satellite lesion and MVI were currently acceptable risk factor associated with HCC patients' prognosis.

The relationship between systematic inflammation, such as PLR and NLR, and cancerous prognosis has been universally accepted by many investigators. Elevated NLR and PLR were identified as independent prognostic factors [21, 23] The systemic inflammatory response such as NF-κB pathway, might lead to aberrant release of pro-inflammatory cytokines and inflammatory mediators, promoting the tumor to proliferate and metastasize via the promotion of angiogenesis, DNA damage, and apoptosis inhibition. [24] In the current study, we defined the mean value of PLR and NLR as cutoff value, and NLR (> 3) were demonstrated as independent risk factor. Elevated NLR got the score of appropriate 12.7 points for OS prediction and 11.7 points for RFS prediction. In our nomograms, the predictive power was enhanced when incorporating these risk predictors, with index of 0.767 for OS prediction and 0.746 for RFS prediction.

According to our nomogram, each individual could achieve a predicted 3- and 5-year OS and RFS after hepatectomy, which cannot be realized by the conventional staging systems placing patients into prognostic groups. For example, BCLC system simply stratified the patients into three groups. For the other three systems, they might not distinguish the prognosis between some stages in the training or validation set. Notably, our nomograms could pay sufficient attention to pathological information, such as MVI and satellite lesion. [9, 34, 35] Moreover, in current study, we firstly incorporated the systematic inflammation into a model to predict the prognosis since this simple and effective blood marker could predict various cancerous prognosis. Consequently, our nomogram showed good calibration and discriminatory abilities with C-index value of 0.767 higher than that of previous nomogram(c-index:0.62) and four conventional staging systems [36].

In conclusion, our nomogram focus on tumor itself and imflammation status,which better predicted the individual prognosis after hepatectomy than conventional staging system. Nomogram is relatively easy to read with a simple graphic. When more HCC patients underwent hepatectomy, these nomograms are effective and practical.

The current study had several limitations. Firstly, while the nomogram was internally validated using bootstrapped calibration and validation set, multicenter studies are needed to externally validate the proposed nomogram. Secondly, our nomogram could only apply those with HCC after hepatectomy. Since hepatectomy provides the hope of curative therapy, it is necessary to accurately predict the prognosis of these patients and thus to guild the postoperative therapy. In the future, since more risk factors were indentified, a model with higher accuracy may be established. Thirdly, in the validation set, the sample size was relative small. The proposed nomograms needed to be validated in large sample size and prospective study.

## MATERIALS AND METHODS

### Patients

Patients were identified from a prospectively maintained database of HCC treated with hepatectomy at West China Hospital from July 1, 2007 through July 31, 2014. A cohort of 777 HCC patients were randomly with a 4:1 ratio allocated into the training set and the validation set via SPSS 20.0 software. The training set consisted of 618 patients and the validation set consisted of 159 patients. The diagnoses of HCC were all histologically confirmed based on analysis of paraffin-embedded tissue by experienced liver pathologists in West China Hospital. Clinicopathological variables including age, gender, hepatitis B virus surface antigen (HBsAg), liver cirrhosis, a- AFP, tumor size, number, major vascular invasion, MVI, satellite lesions, differentiation were obtained from our database. The cutoff value of AFP was defined as previous study described. [32] The presence of MVI, visible only on microscopy, was described as determined from histopathological reports, while major vascular invasion were described as tumor invading the portal or/and hepatic vein which can be detected by imaging study or surgical findings. Two or more HCCs that were reported separately with a full description of histopathologic features were described as multiple HCCs. Nodules (< 2 cm) close to the main tumor were described as satellite lesions in the original pathology report. [37] The types of hepatectomy including minor (1 segment), major (2–3 segments) and extended liver resection (> 3 segments) were determined according to the tumor location and size, liver functional reserve, cirrhosis, and estimated volume of future liver remnant based on CT data. All the patients were strictly stratified by BCLC system [38], the seventh AJCC system [39], CLIP system [40] and HKLC system [41]. The inclusion criteria were as follows: (1) without extrahepatic metastasis; (2) HCC treated by hepatectomy; (3) exact diagnosis of pathologically proven HCC; (4) HCC patients should have child-pugh status A, or child-pugh status B which can be improved to A. (5) liver tumor can be completely removed (R0), and for patients with liver cirrhosis, the residual liver volume should be at least 40% of total liver volume. The exclusion criteria were as follows: (1) with other cancers; (2) with severe cardiovascular and pulmonary disease; (3) the major vascular invasion was involving in the trunk of portal vein or the inferior vena cava; (4) imaging study (liver ultrasound or abdominal contrast-enhanced computed tomography (CT) scan suggests severe liver cirrhosis or/and ascites (5) incomplete clinicopathological reports and follow-up data. Informed consent for using their data in research was obtained from all patients, and the study protocol was approved by the Ethic Committee of West China Hospital.

### Follow-up

The median follow-up for our cohort was 33.13 months in the training set and 34.80 months in the validation set. All of the patients were regularly followed at first month and every 3 months after surgery. Routing blood tests, liver function tests, AFP levels measurements, HBV-DNA levels, liver ultrasound were performed at each follow up. Once HCC recurrence including intrahepatic or/and extrahepatic recurrence was suspected, CT or/and magnetic resonance imaging (MRI) was chosen to conform the lesions, and chest CT scan and bone scan were necessary. After HCC recurrence, Patients were evaluated at multidisciplinary team (MDT) in West China Hospital for treatment guidance based on the status of tumor and general condition. The MDT mainly comprised of experienced hepatic surgeon, radiologists, pathologists and oncologists in West China Hospital. Liver transplantation, resection, ablation, and TACE and palliative therapy could be applied. Patients were administrated anti-virus therapy, such as Entecavir (0.5 mg/day), if their HBV-DNA levels were > 1.00E + 03 copies/ml before and after surgery during follow up. RFS and OS were calculated from the date of initial treatment until the date of detection of recurrence and death or the date of last follow-up.

### Statistical analysis

Continuous variables were displayed as mean ± standard deviation and compared by Student's *t* test or Mann-Whitney *U* test (non-normal distribution data). Categorical data were shown as frequency and assessed by Fisher's exact test and two-tailed χ^2^ test. Survival curves stratified by various stage systems were evaluated using the Kaplan-Meier method and compared using the log-rank test.

Potential risk factors significant in univariate analysis were included multivariate analysis with forward step-wise selection process. We subsequently used the risk factors from multivarate analysis of the training set to construct a nomogram. Harrell's concordance index (c-index) ranging from 0.5 (randomness) to 1 (perfect discrimination) was used to evaluate the concordance between predicted and actual observed responses of patients. [42] Being concordant means the nomogram assigned a higher probability of death (alive) to the patient who died (alive) than the one alive (death). The c-index is the probability of being concordant out of all predicted and actual patient pairs. [43].

Calibration plots were generated to investigate the performance characteristics of our nomogram at 3 and 5 years after surgery in the training set and the validation set. Nomogram was internally validated using bootstrapping with 1000 resamples. Moreover, we estimate the predictive performance when the models are applied to new patients in the validation set. The predict power between the nomogram and other four current staging systems in the training set and the validation set were assessed using the rcorrp.cens package in Hmisc in R and were shown as the C-index. A higher C-index indicated a higher predict power for HCC. A *P* < 0.05 was considered statistically significant. All analyses were carried out using R version 3.3.0 with the rms packages (http://www.R-project.org) and SPSS version 20.0 (Chicago, IL, USA).
